# Identification and validation of an autophagy-related gene signature for predicting prognosis in patients with esophageal squamous cell carcinoma

**DOI:** 10.1038/s41598-022-05922-4

**Published:** 2022-02-04

**Authors:** Xiaobo Shi, You Li, Shupei Pan, Xiaoxiao Liu, Yue Ke, Wei Guo, Yuchen Wang, Qinli Ruan, Xiaozhi Zhang, Hongbing Ma

**Affiliations:** 1 Department of Radiation Oncology, The Second Affiliated Hospital of Xi’an Jiaotong University, Xi’an, China; 2 Department of Peripheral Vascular, The First Affiliated Hospital of Xi’an Jiaotong University, Xi’an, China; 3 Department of Radiation Oncology, The First Affiliated Hospital of Xi’an Jiaotong University, Xi’an, China

**Keywords:** Cancer genomics, Cancer models, Oesophageal cancer

## Abstract

Esophageal squamous cell carcinoma (ESCC) is the main subtype of esophageal cancer. Since autophagy-related genes (ARGs) play a key role in the pathogenesis of many tumors, including ESCC, the purpose of this study is to establish an autophagy-related prognostic risk signature based on ARGs expression profile, and to provide a new method for improving prediction of clinical outcomes. We obtained the expression profiles of ESCC from public data (GSE53625) and extracted the portion of ARGs. Differential expression analysis and enrichment analysis were performed to confirm abnormal autophagy-related biological functions. Univariate and multivariate Cox regression analyses were performed on RNA microarray data (GSE53625) to construct a prognostic risk signature associated with autophagy. The performance of the model was evaluated by receiver operating characteristic (ROC) analysis, survival analysis and Brier score. The model was subjected to bootstrap internal validation. The potential molecular mechanism of gene signature was explored by gene set enrichment analysis (GSEA). Spearman correlation coefficient examined the correlation between risk score and immune status and ferroptosis. The expression levels of genes and proteins were validated by qRT-PCR and immunohistochemistry in ESCC cell lines and ESCC tissues. We constructed and validated an autophagy-related prognostic risk signature in 179 patients with ESCC. The long-term survival of patients in high-risk group was lower than that in low-risk group (log-rank, *P* value < 0.001). ROC analysis and Brier score confirmed the reliability of the signature. GSEA results showed significant enrichment of cancer- and autophagy-related signaling pathways in the high-risk ESCC patients and immunoregulatory signaling pathways in the low-risk ESCC patients. Correlation analysis showed that the risk signature can effectively predict the effect of immunotherapy. About 33.97% (71/209) ferroptosis-related genes were significantly correlated with risk scores. Finally, the results of qRT-PCR and immunohistochemistry experiments were consistent with bioinformatics analysis. In brief, we constructed a novel autophagy-related gene signature (VIM, UFM1, TSC2, SRC, MEFV, CTTN, CFTR and CDKN1A), which could improve the prediction of clinical outcomes in patients with ESCC.

## Introduction

Esophageal cancer is one of the most common malignancies and mainly includes two pathological subtypes: squamous cell carcinoma and adenocarcinoma^1^. Esophageal squamous cell carcinoma (ESCC) is the most dominant pathological type in China, accounting for almost 90% of all cases of esophageal cancer^2^. Despite great advances in diagnosis and treatment strategies for ESCC over the past few decades, the 5-year survival rate is still poor, only about 15–25%^3–5^. Hence, the development of new effective prognostic biomarkers to guide ESCC treatment is imperative.

Autophagy is a process in which cells use lysosomes to degrade damaged, denatured or senescent macromolecular substances and organelles^6^. The relationship between autophagy and tumor is very complex and plays a dual role in tumorigenesis, maintenance, and tumor progression. In the process of tumor initiation and malignant transformation, inhibition of autophagy can promote the growth of tumor cells, indicating that autophagy plays a role in tumor inhibition. However, in the process of tumor progression, autophagy can play a protective mechanism by promoting tumor cell metastasis and inhibiting tumor cell apoptosis^7^. It has been reported that autophagy is related to the diagnosis and treatment of esophageal squamous cell carcinoma^8,9^; however, the relationship between autophagy and ESCC has not been fully revealed. Hence, it is of great significance to search for biomarkers related to autophagy for individualized treatment and prognosis of ESCC patients.

In this study, we comprehensively analyzed the expression profile of autophagy-related genes (ARGs) in 179 patients with ESCC to construct and validate an autophagy-related prognostic risk signature. With this novel risk signature, patients with ESCC were divided into high-risk group and low-risk group, and there was significant difference in overall survival time (OS). The potential molecular mechanism of gene signature was explored by gene set enrichment analysis (GSEA). Importantly, we also validated ARGs expression using ESCC cell lines, ESCC tissues and multiple databases to ensure the accuracy and replicability of bioinformatics results. We aim to give more helpful guidance for individualized treatment and prognosis of ESCC patients.

## Materials and methods

### Data collection and preparation

RNA expression profiles and corresponding clinical information in tumor and normal tissues of 179 ESCC patients are publicly available (GSE53625)^10^. The GSE53625 dataset contained normalized gene expression data, but the batch effect was still present, so the “limma” package was used to remove the batch effect (Supplementary Fig. 1)^11^. The maximum expression was considered to be the expression of genes with several probes. 232 and 572 autophagy-related genes were searched from Human Autophagy Database (HADb, http://www.autophagy.lu/) and Molecular Signatures Database (version 7.2, http://www.gsea-msigdb.org/gsea/index.jsp), respectively. Finally, the expression levels of 585 ARGs were obtained from GSE53625 dataset (Supplementary Table 1).

### Differentially expressed analysis of ARGs

By comparing paired tumor to normal tissues from 179 ESCC patients, differentially expressed autophagy-related genes (DE-ARGs) were identified by absolute value of log2 fold change (log2FC) > 1 and false discovery rate (FDR) < 0.05.

### Functional enrichment analysis

In order to explore the potential mechanism of DE-ARGs affecting ESCC, the “clusterProfiler” package was used to analyze the function and pathway enrichment of gene ontology (GO) and Kyoto Encyclopedia of Genes and Genomes (KEGG, www.kegg.jp/kegg/kegg1.html)^12–17^.

### Construction of autophagy-related prognostic risk signature

All ARGs were analyzed by univariate Cox regression analysis, and genes with *P* < 0.05 were defined as prognosis-related genes. Then, multivariate Cox analysis was performed based on the Akaike Information Criterion (AIC) to establish the optimal prognostic risk model. Furthermore, the ARGs-related prognostic model was established by multiplying multivariate Cox regression coefficients by the expression level of each variable.

### Validation and assessment of the prognostic signature

The risk score of ESCC patients was calculated by using the prognostic signature. According to the median risk score, all ESCC patients were separated into high-risk group and low-risk group. The difference of overall survival between the two groups was analyzed by Kaplan–Meier survival curve. Receiver operating characteristic (ROC) curve and area under ROC (AUC) were used to evaluate the sensitivity and specificity of the prognostic signature. The Brier score and calibration curves were used to assess the calibration of the prognostic model.

In order to verify the effectiveness of the prognostic model, the bootstrap method based on 1000 resampling was used to obtain the testing set^18,19^. The training set was the original dataset. In the testing set, AUC and Brier score for the prognostic model were calculated using the R package of the “riskRegression”. The relationship between the risk score and clinical factors was also analyzed to identify the validity of the risk signature, and the survival prediction ability of prognostic factors was further compared. By comparing the clinical traits, univariate and multivariate Cox regression analyses were used to confirm the independence of the prognostic model. At the same time, a nomogram was constructed using the Cox regression coefficients with the R package “rms”, and its calibration curves were drawn.

### Gene set enrichment analysis

GSEA, using the “clusterProfiler” package, was employed for assessing the possible mechanisms between high-risk and low-risk groups of ESCC patients based on the KEGG (v7.2) and Hallmark (v7.2) gene sets collections.

### Relationship between the risk score and immune status and ferroptosis

The CIBERSORT algorithm was employed to calculate the relative proportion of 22 tumor-Infiltrating Immune Cells (TICs) in 179 ESCC samples^20,21^. After quality screening (*P* < 0.05), 107 cases of ESCC could be for subsequent analysis. The Spearman coefficient examined the correlations between risk score and 22 TICs proportion. Meanwhile, Spearman correlation was also applied to detect the relationship between the risk score and immunotherapy-related targets, such as programmed cell death 1(PDCD1, also known as PD-1) and programmed cell death 1 ligand 1(PD-L1, also known as CD274).

Ferroptosis is a newly introduced form of programmed cell death discovered in recent years. Accumulating studies have revealed ferroptosis could inhibit tumor growth or promote tumor proliferation in tumor development, and there is crosstalk with autophagy at the molecular level^22,23^. Then, Spearman correlation examined the correlation between the risk score and ferroptosis -associated genes, which were identified from the FerrDb website (http://www.zhounan.org/ferrdb/index.html).

### Multiple database validation

To minimize the bias, the expression of ARGs in the prognostic signature was detected using the following databases: The Cancer Genome Atlas (TCGA) (http://cancergenome.nih.gov/), Oncomine^24^ and Cancer Cell Line Encyclopedia^25^. RNA-seq data files from TCGA ESCC samples were analyzed as previously described^26^. Kaplan–Meier plotter database (http://www.kmplot.com/) was utilized to validate the prognosis of critical ARGs in the prognostic signature^27^.

### qRT-PCR analysis

Human ESCC cell lines (Eca-109, KYSE-150 and TE-1) were purchased from the Cell Bank of the Chinese Academy of Sciences (Shanghai, China). Human normal esophageal cell line (Het-1A) was purchased from ATCC (American Type Culture Collection, Manassas, USA). Cell culture, total RNA extraction and qRT-PCR analysis were carried out as described previously^28^. The relative expression of each gene in prognostic signature was calculated after normalization to GAPDH. Primer sequences are listed in Supplementary Table 2.

### Immunohistochemistry (IHC) analysis

Paraffin-embedded ESCC tissues (n = 42) were obtained from pathologically proved ESCC patients who received esophagectomy in the First Affiliated Hospital of Xi'an Jiaotong University from January 2010 to January 2011. In addition, 10 cases of paraffin-embedded normal esophageal mucosa were used as controls. The experiments, including any relevant details, were approved by the Ethics Committee of the First Affiliated Hospital of Xi’an Jiaotong University. The study was performed in accordance with relevant guidelines and regulations. Verbal informed consent was obtained from the patient(s) for their anonymized clinical information to be published in this article. The detailed clinical data of ESCC patients are supplied in Supplementary Table 3. IHC staining analysis was conducted as described previously^29^. The primary antibody used was anti-cortactin (CTTN) (ab81812, Abcam, 1:300).

### Statistical analysis

Statistical analyses were performed in R software (Version 4.0.2; https://www.R-project.org)^30^. Statistical significance was set at *P*-value < 0.05. The Kaplan–Meier method was used for survival analysis, and the log-rank test was used to compare the results. Wilcoxon test was used for two independent samples or the paired data. One-way analysis of variance (ANOVA) or Kruskal–Wallis test was used to test the difference among multiple groups. *P* < 0.05 and Benjamini–Hochberg adjusted *P* < 0.05 were considered as the cut-off criterion for enrichment analysis.

### Ethics statement

The work was approved by the Ethics Committee of The First Affiliated Hospital of Xi'an Jiaotong University.

## Results

### Identification of 87 differentially expressed ARGs

**Figure 1:**
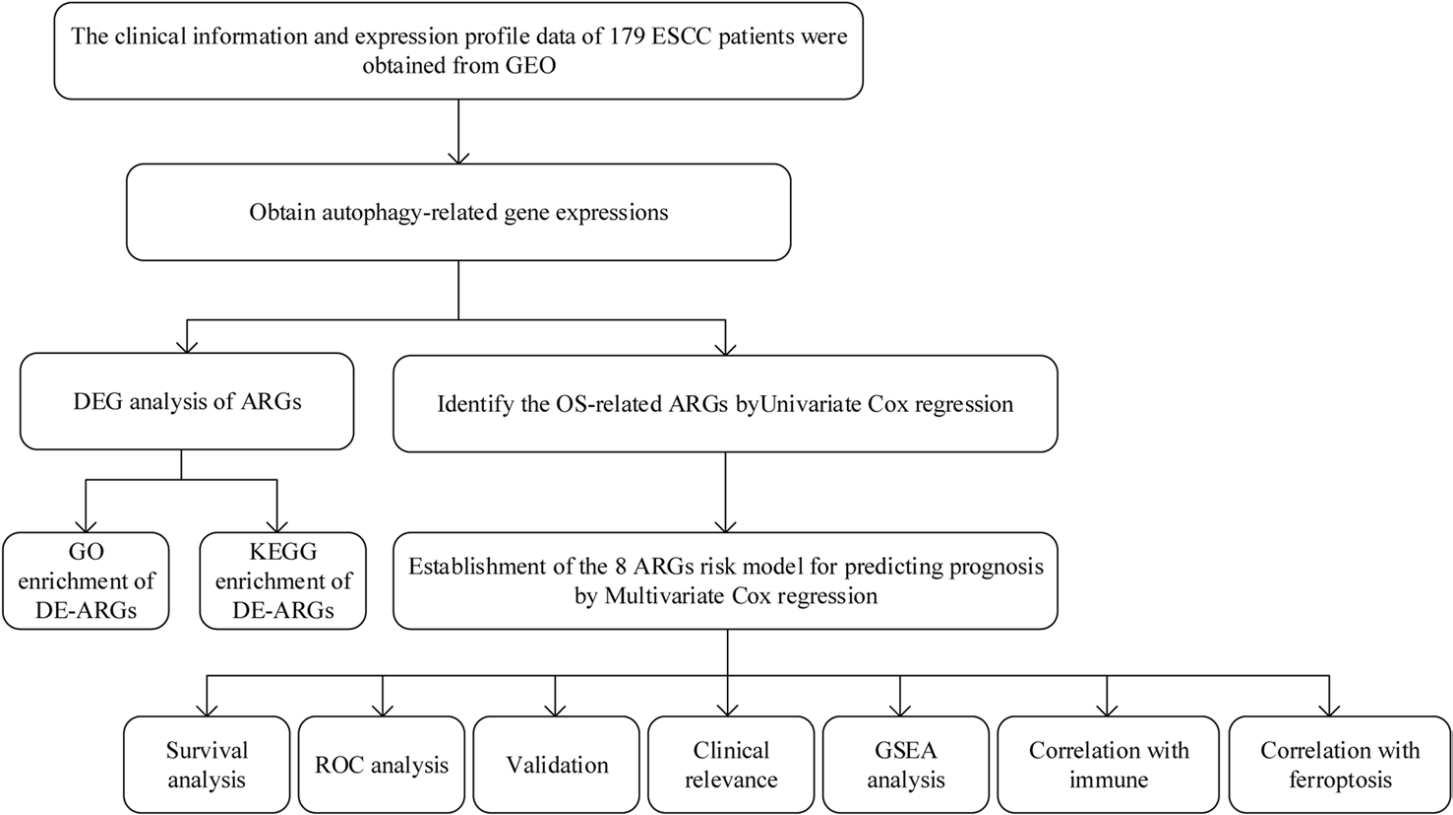
Flow diagram of the overall procedures.

**Figure 2:**
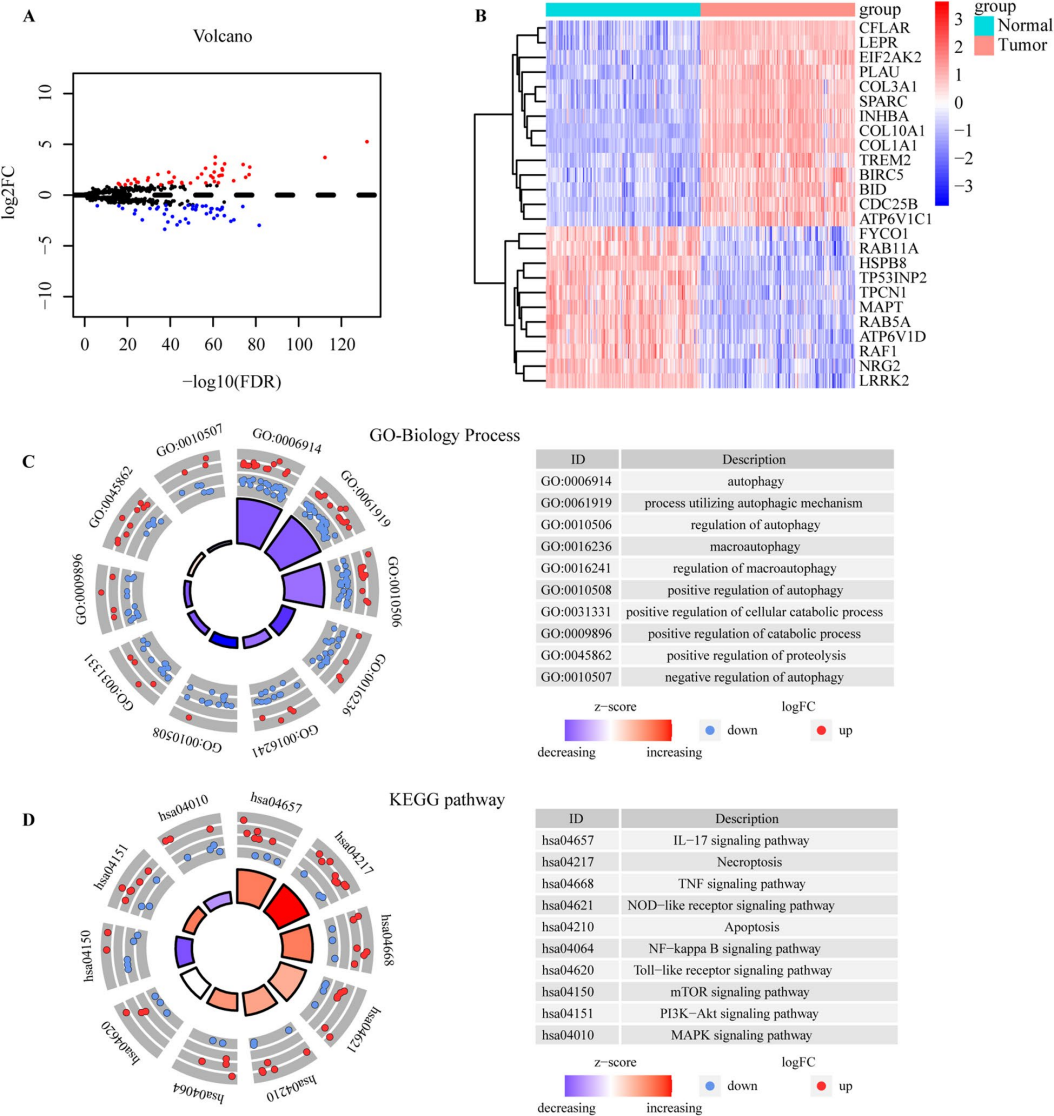
Autophagy-related gene screening and enrichment analysis. (**A**) Volcano plot of differentially expressed autophagy-related genes (DE-ARGs) between tumor tissues and normal tissues. Red dots indicate upregulated genes, while blue dots indicate downregulated genes with statistical significance. (**B**) The heatmap of top 25 DE-ARGs between tumor tissues and normal tissues. (**C**, **D**) Gene ontology (GO) and Kyoto Encyclopedia of Genes and Genomes (KEGG) enrichment analysis. BP stands for biological process. The higher the Z-score value indicated, the higher expression of the enriched pathway. The figures were created using R software v4.0.2.

Figure 1 presents the flow diagram of the research. According to the criteria for screening differential genes, the expression levels of 87 of the 585 ARGs in ESCC were significantly changed compared with the normal controls, among which 44 and 43 ARGs were respectively up-regulated and down-regulated. Volcano and heatmap plots are shown (Fig. 2A, B). GO biological process enrichment analysis of 87 DE-ARGs (Supplementary Table 4) showed that they were mainly involved in the regulation of autophagy and catabolic processes (Fig. 2C). KEGG analysis showed that these genes were mainly involved in the IL-17 signaling pathway, Necroptosis, TNF signaling pathway and apoptosis signaling pathway (Fig. 2D). The enrichment analysis details are given in Supplementary Table 5.

### Construction and validation of the autophagy-related gene signature

**Figure 3:**
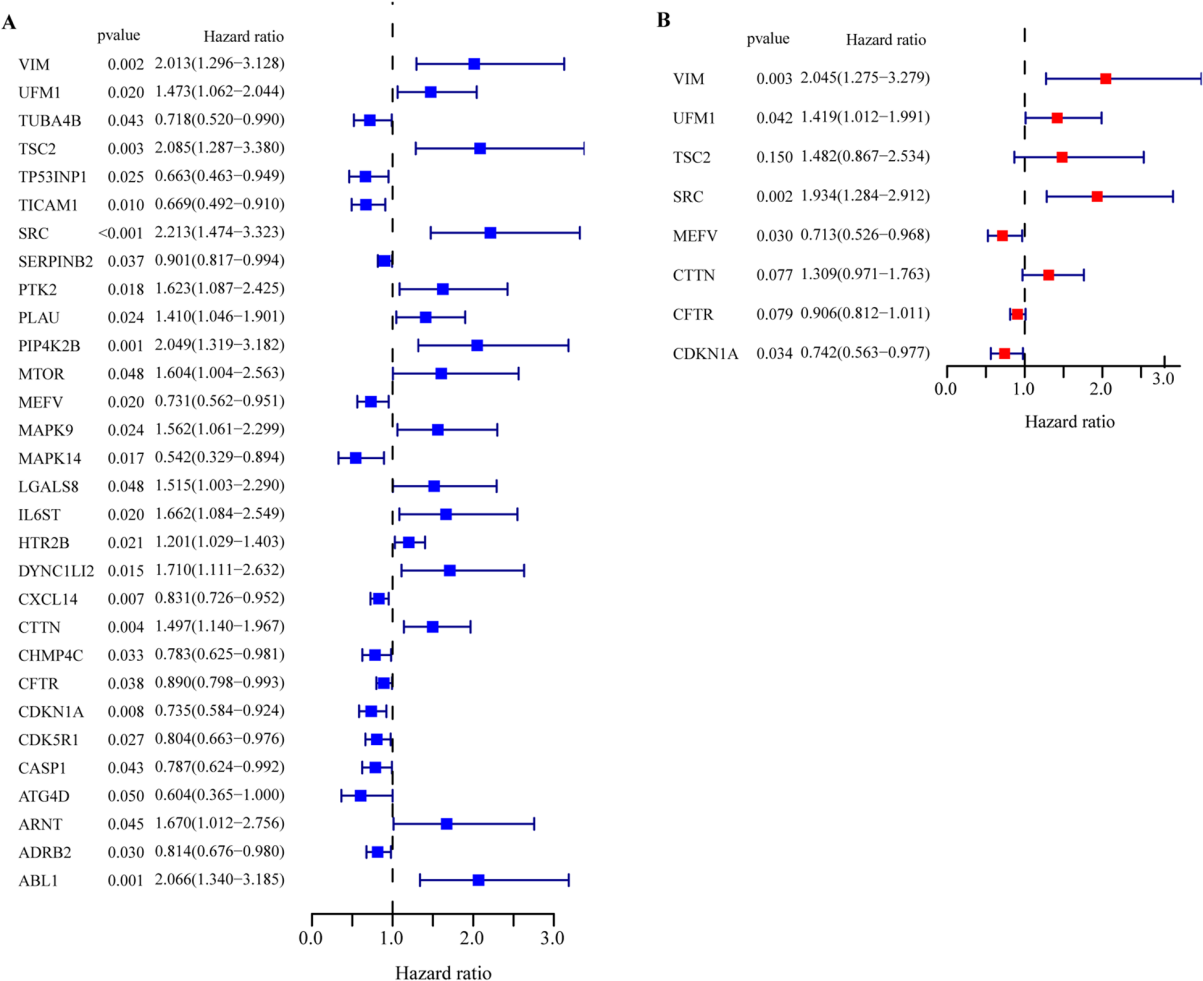
The process of cox regression model construction. (**A**) The forest plot of univariate cox regression identified 30 autophagy-related genes (ARGs) associated with overall survival (OS). (**B**) The forest plot of multivariate Cox regression analysis of 8 ARGs associated with OS.

Univariate Cox regression analysis of all ARGs (rather than DE-ARGs) revealed 30 prognostic genes in ESCC (*P* < 0.05) (Fig. 3A, Supplementary Table 6). Subsequently, the prognostic signature based on 8 ARGs was established using stepwise multivariate Cox regression analysis (Fig. 3B, Supplementary Table 7). The formula for the prognostic model was given as follow: Risk score = [0.71520 × expression level of VIM] + [0.35023 × expression level of UFM1] + [0.39370 × expression level of TSC2] + [0.65958 × expression level of SRC] + [—0.33759 × expression level of MEFV] + [0.26911 × expression level of CTTN] + [− 0.09856 × expression level of CFTR] + [− 0.29849 × expression level of CDKN1A].

**Figure 4:**
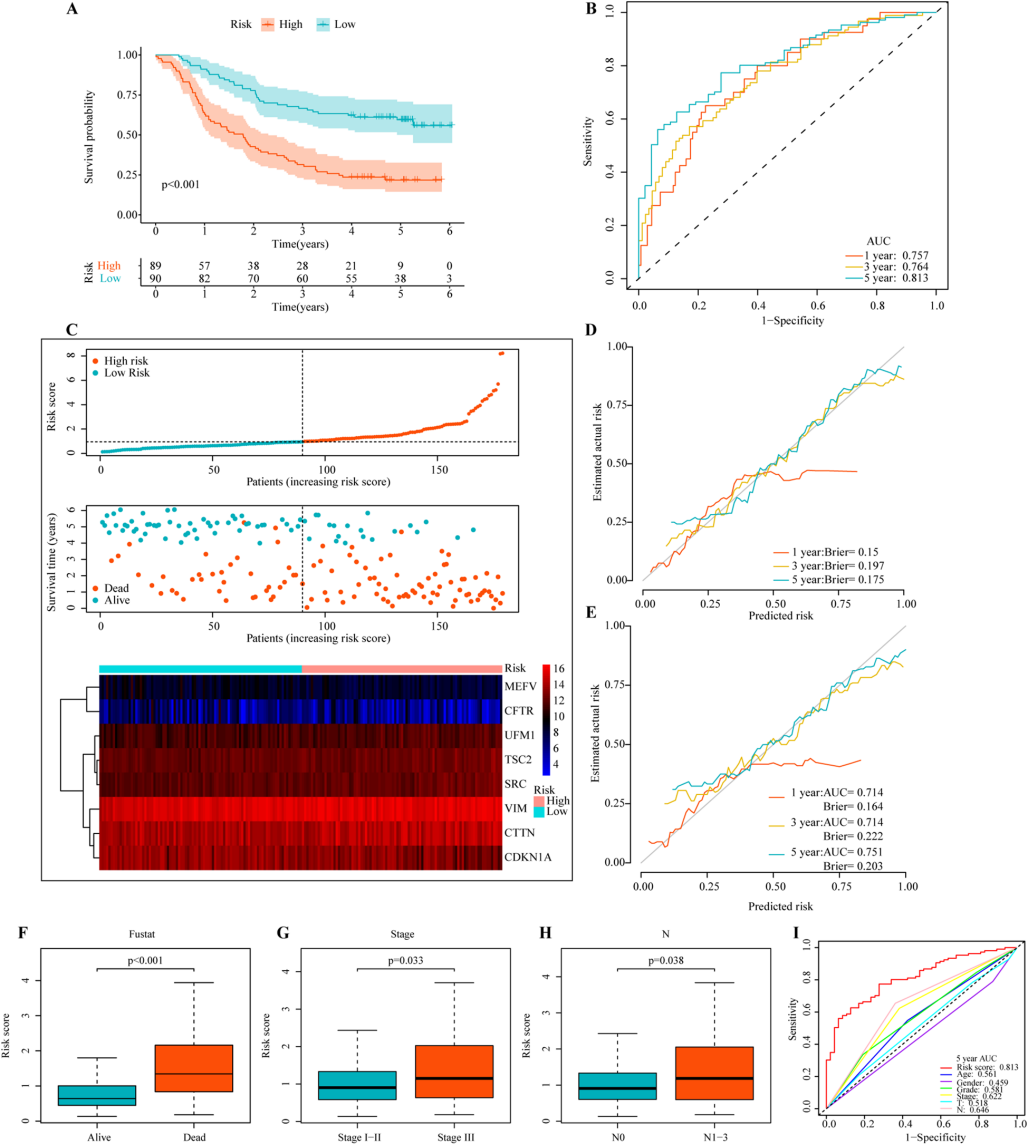
The prognostic value of risk model of the 8 autophagy-related genes in 179 ESCC patients. (**A**) Kaplan–Meier survival analysis for ESCC patients in the training dataset. (**B**) Time-dependent ROC curve analysis for ESCC patients in the training dataset. (**C**) Autophagy-related genes risk score analysis (risk scores distribution of the ESCC patients; survival status and duration of the ESCC patients; Heatmap of the 8 genes expression). (**D**) The calibration curves with brier scores for predicting the 1-, 3- and 5-year OS in the training dataset. (**E**) The calibration curves with AUCs and brier scores for predicting the 1-, 3- and 5-year OS in the testing dataset. (**F**–**H**) Clinical significance of the prognostic signature of ESCC (survival outcome, tumor stages and N stage). (**I**) The ROC analysis of 5-year OS for the signature and the clinicopathologic parameters. The figures were created using R software v4.0.2.

The risk score of ESCC patients was calculated by using the prognostic signature. According to the median risk score, all ESCC patients were separated into high-risk group (n = 89) and low-risk group (n = 90). The long-term survival of patients in high-risk group was lower than that in low-risk group (log-rank, *P* < 0.001) (Fig. 4A). The sensitivity and specificity of prognostic signature were evaluated by ROC curves of 1-, 3- and 5-year OS rates, with AUCs of 0.757, 0.764 and 0.813, respectively (Fig. 4B). The distribution of risk scores, patients’ survival status and the expression values of the eight genes are shown in Fig. 4C.

The calibration curves of prognosis signature for the prediction of 1-, 3- and 5-year OS rates demonstrated good agreement in the training set, with Brier scores of 0.15, 0.197, and 0.175, respectively (Fig. 4D). Through bootstrapping verification, the AUCs and brier scores of predicted 1-, 3- and 5-year OS rates were 0.714, 0.714, 0.751, 0.164, 0.222 and 0.203, respectively, indicating that the model had good discrimination and calibration (Fig. 4E).

### Independence of the autophagy-related genes signature for ESCC patients

The analysis of the risk score and clinicopathological features showed that the risk score was associated with survival outcomes (*P* < 0.001), stage (*P* = 0.033) and N stage (*P* = 0.002) (Fig. 4F-H, Supplementary Fig. 2). Furthermore, Fig. 4I showed the 5-year AUC value of the prognostic signature was significantly higher than that of age (AUC = 0.561), gender (AUC = 0.459), grade (AUC = 0.581), stage (AUC = 0.622) and T stage (AUC = 0.518) and N stage (AUC = 0.646).

**Figure 5:**
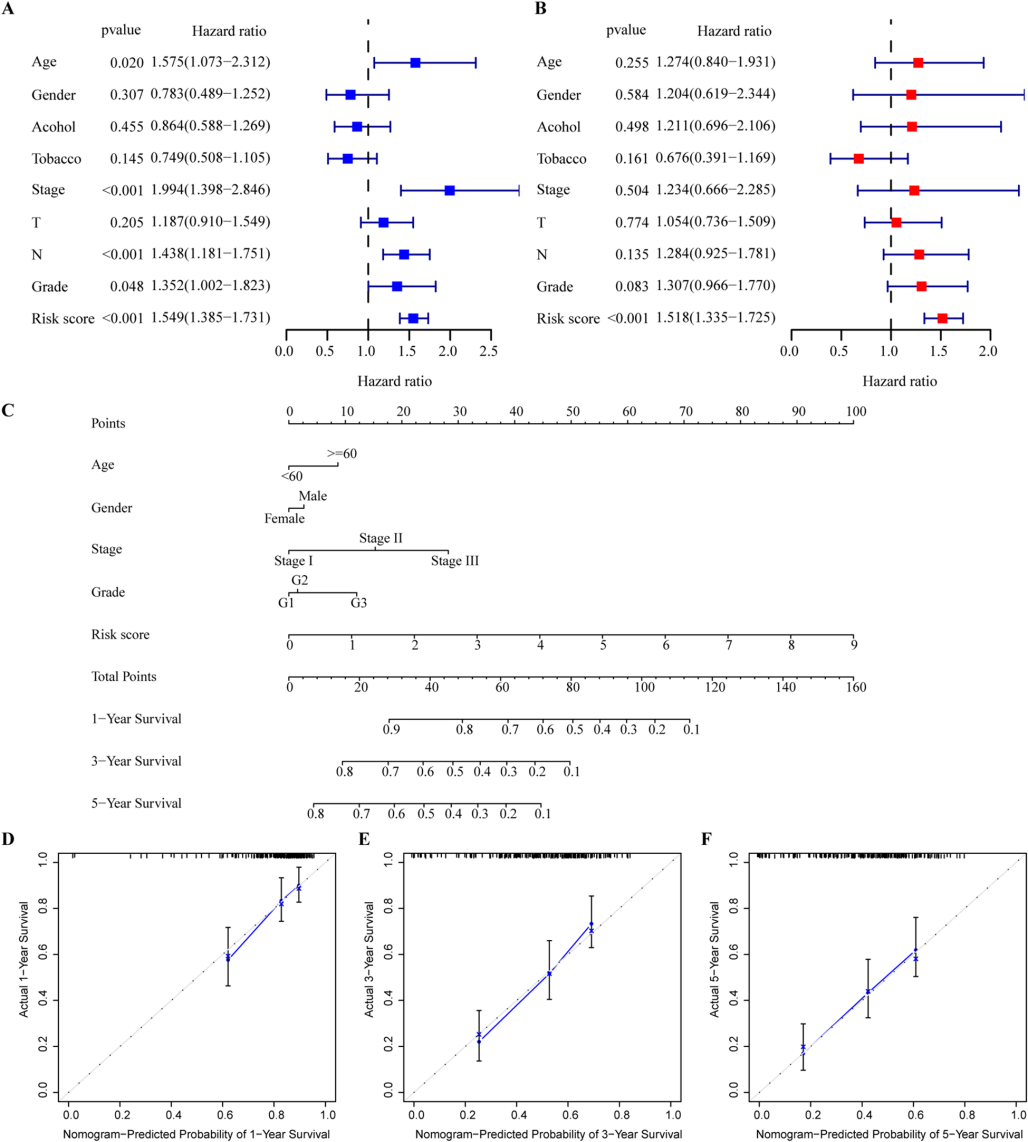
Independent prognostic analysis of risk scores and clinical parameters. (**A**) The univariate and (**B**) multivariate Cox regression analysis of the associations between the risk scores and clinical parameters and the overall survival (OS) of ESCC patients. (**C**) Nomogram constructed by combining clinical characteristics and the risk score. (**D**–**F**) The calibration curves for predicting 1-,3-, and 5-years OS. Age was analyzed as a categorical variable: age ≥ 60 = 1, age < 60 = 0; Stage, T stage, N stage, grade, and risk score were continuous variable. Specifically, Stage: I = 1, II = 2, III = 3; T stage: T1 = 1, T2 = 2, T3 = 3, T4 = 4; N stage: N0 = 0, N1 = 1, N2 = 2, N3 = 3; Histologic grade: G1 = 1, G2 = 2, G3 = 3.

Univariate Cox regression showed that advanced age, higher tumor stage, N stage, grade, and higher risk score were risk factors for poor prognosis (Fig. 5A). Meanwhile, multivariate Cox regression analysis revealed that the autophagy-related genes signature was an independent prognostic factor for ESCC patients, whereas the clinicopathologic features were not (Fig. 5B). To develop a quantitative method for predicting the prognosis of ESCC patients, we constructed a prognostic nomogram based on the risk score of autophagy and clinicopathological features, such as age, gender, stage and grade (Fig. 5C). The calibration curves for 1-, 3-, and 5-years OS showed excellent agreement between predicted and observed outcomes (Fig. 5D-F).

### Gene set enrichment analysis

**Figure 6:**
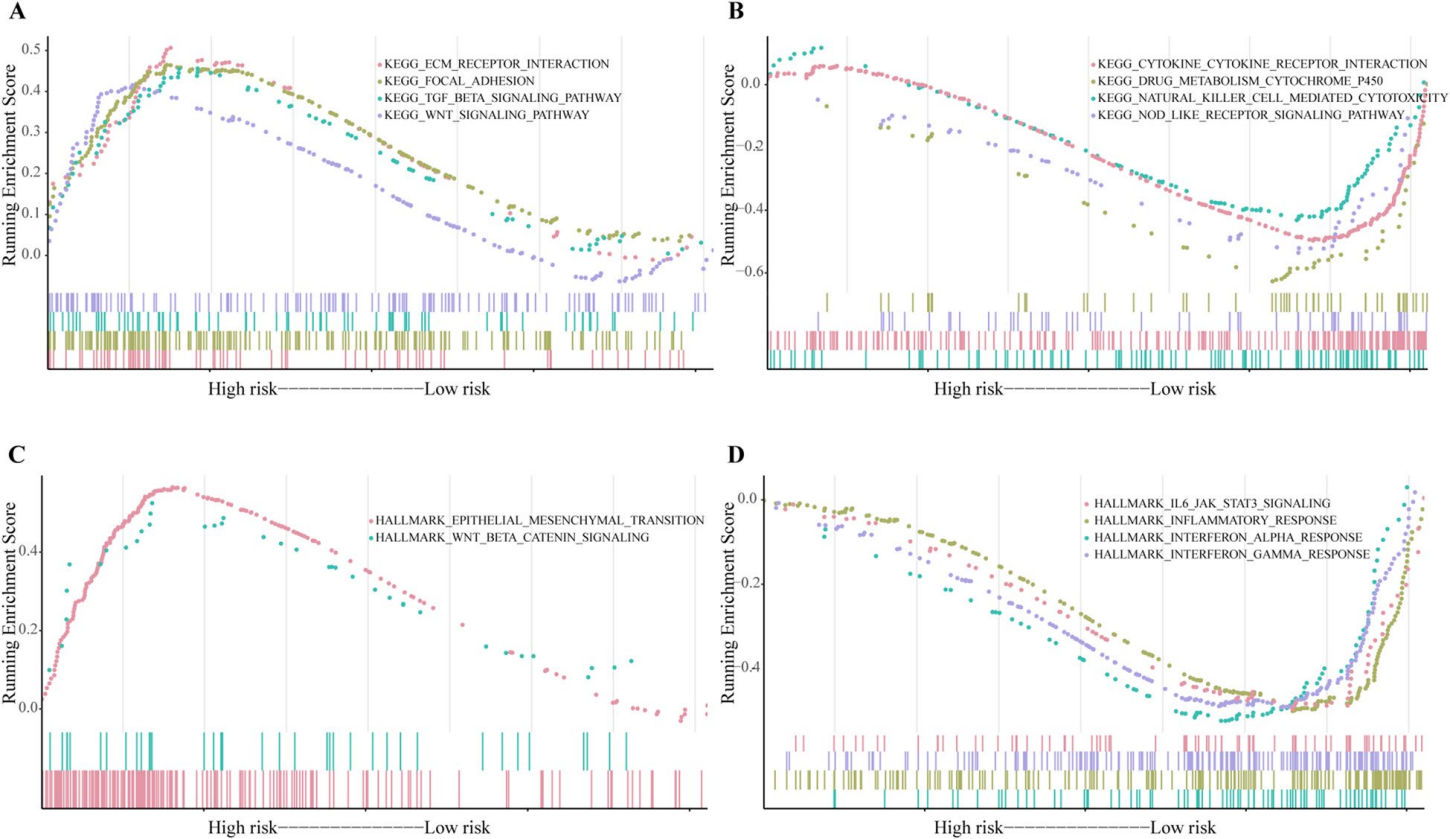
Functional enrichment analysis based on the prognosis model of the 8 autophagy-related genes by GSEA. Some pathways were enriched in the high-risk group (**A**) and low-risk group (**B**) using KEGG pathways. Some pathways were enriched in the high-risk group (**C**) and low-risk group (**D**) using HALLMARK collection.

The GSEA results revealed that cancer- and autophagy-related signaling pathways were significantly enriched in the high-risk ESCC patients, including ECM receptor interaction, WNT signaling pathway, epithelial-mesenchymal transition, focal adhesion and TGF- β signaling pathway (Fig. 6A, C). Meantime, immunoregulatory pathways were significantly enriched in the low-risk ESCC patients, including Natural killer cell mediated cytotoxicity, Cytokine-cytokine receptor interaction, NOD like receptor signaling pathway, Interferon-alpha response, Interferon-gamma response and IL6 JAK STAT3 signaling pathway (Fig. 6B, D). The enrichment analysis details are given in Supplementary Table 8.

### Immune cell infiltration analysis

**Figure 7:**
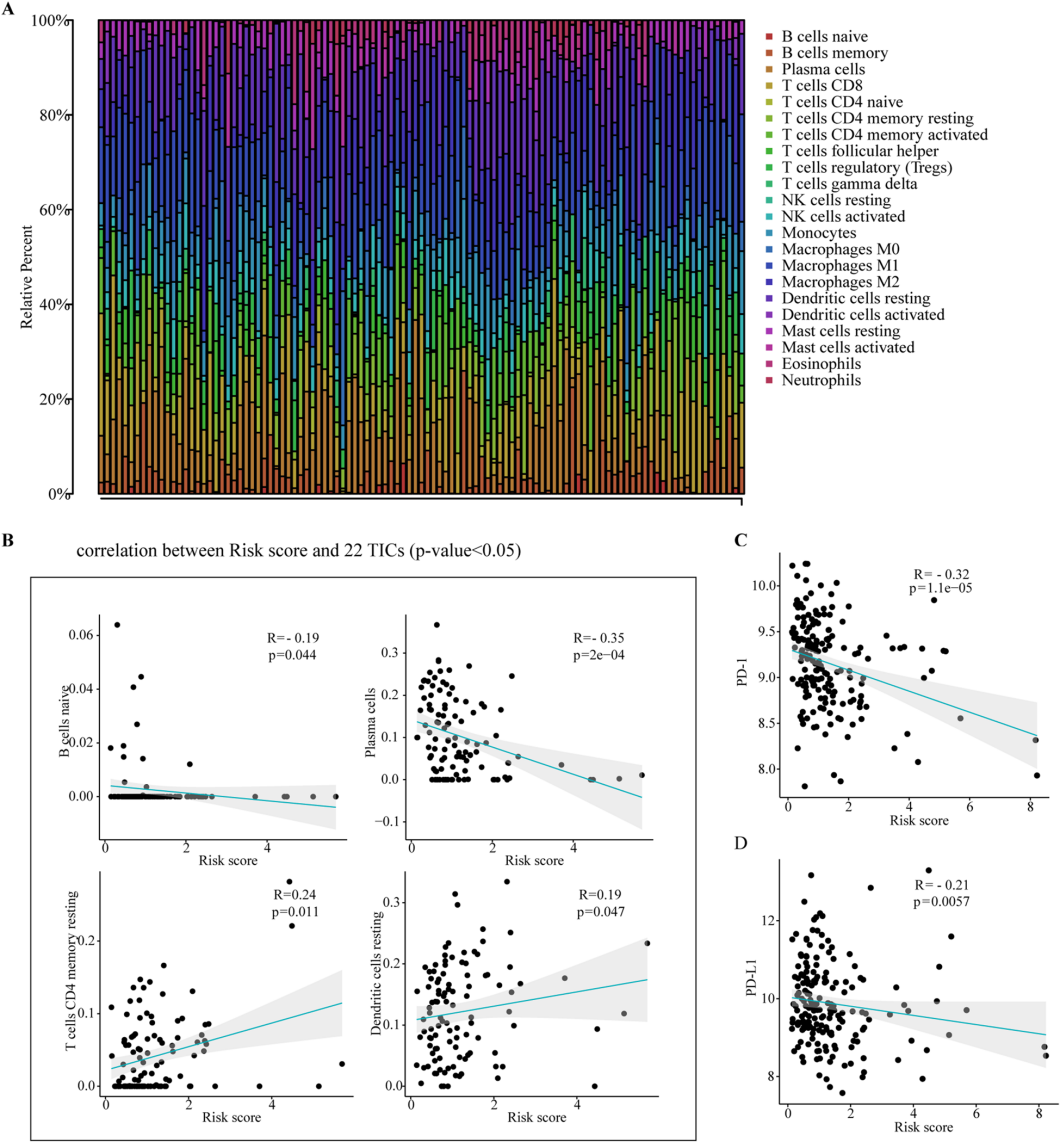
Correlations between the risk score and tumor-Infiltrating Immune Cell (TIC) and expression level of immunotherapy-related targets in ESCC. (**A**) The relative content distribution of 22 TICs of ESCC samples. (**B**) The correlations between the risk score and TICs (only correlations with significate were plotted). (**C**, **D**) The correlations between the risk score and expression level of PD-1and PD-L1. Correlation test is conducted by the Spearman coefficient.

Figure 7A shows the relative content distribution of 22 TICs in the ESCC cohort and their correlation with risk scores. The risk score had negative correlation with the levels of B cells naive (R = − 0.19, *P* = 0.044) and Plasma cells (R = − 0.35, *P* < 0.001). However, the risk score was positively correlated with the levels of T cells CD4 memory resting (R = 0.24, *P* = 0.011) and Dendritic cells resting (R = 0.19, *P* = 0.047) (Fig. 7B). The mRNA expression levels of PD-1(R = − 0.32, *P* < 0.001) and PD-L1(R = − 0.21, *P* = 0.0057) were negatively correlated with the risk score, indicating that patients with lower risk scores might have a better response to PD-L1 immunotherapy (Fig. 7C, D).

### Identification of the ferroptosis correlation with the prognostic signature

**Figure 8:**
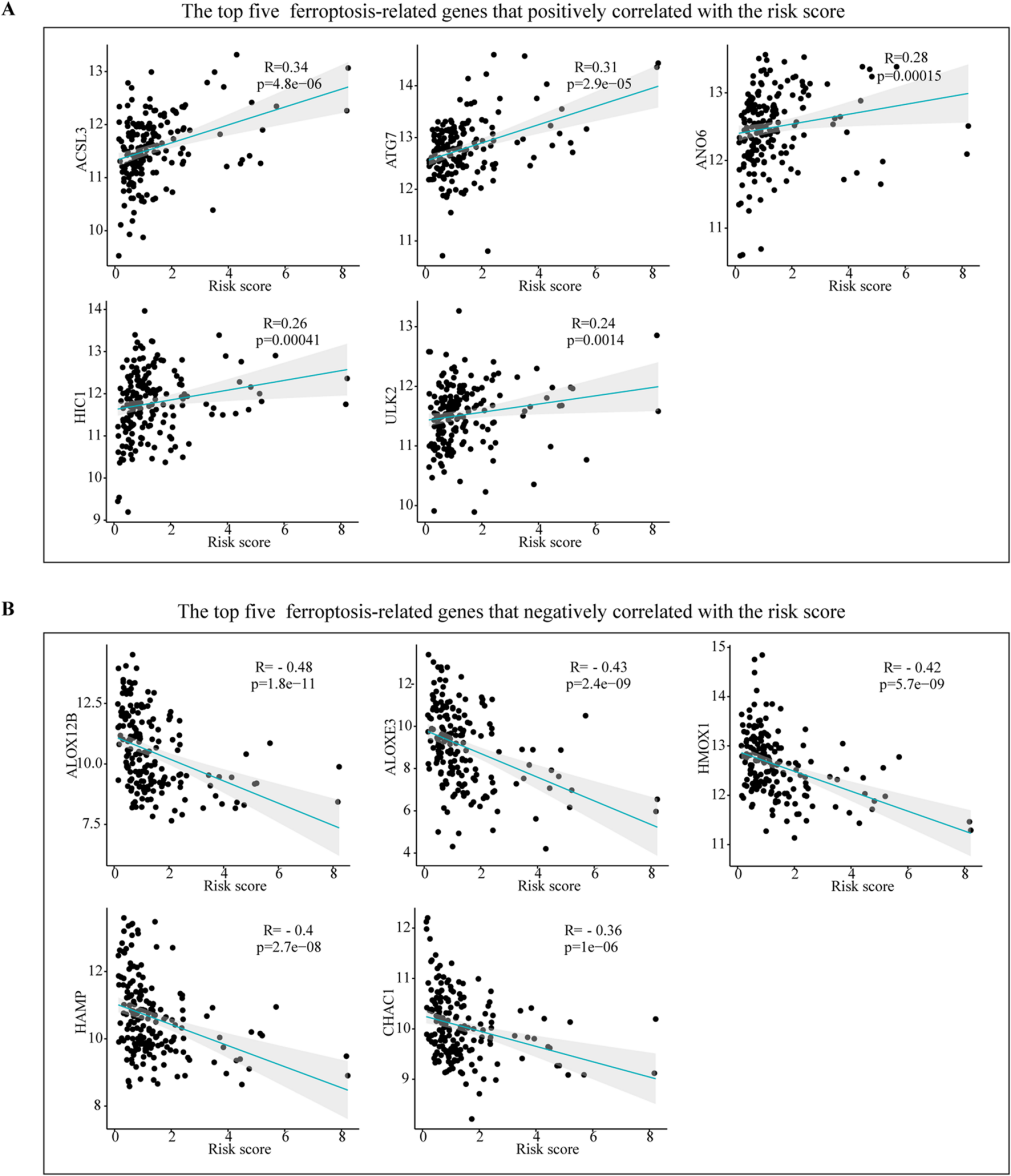
Correlations between the risk score and the ferroptosis-related genes in ESCC. (**A**) The top five positive correlations. (**B**) The top five negative correlations. Correlation test is conducted by the Spearman coefficient.

Among the 209 ferroptosis-related genes, 71 (33.97%) were significantly associated with risk scores, of which 25 were positively correlated with risk scores and 46 were negatively correlated with risk scores (Supplementary Table 9). As shown in Fig. 8, ACSL3, ATG7, ANO6, HIC1and ULK2 are the leading five ferroptosis -related genes that have a positive correlation with the risk score, while the top five ferroptosis -related genes that are negatively correlated with the risk score are ALOX12B, ALOXE3, HMOX1, HAMP and CHAC1.

### Multiple database validation

**Figure 9:**
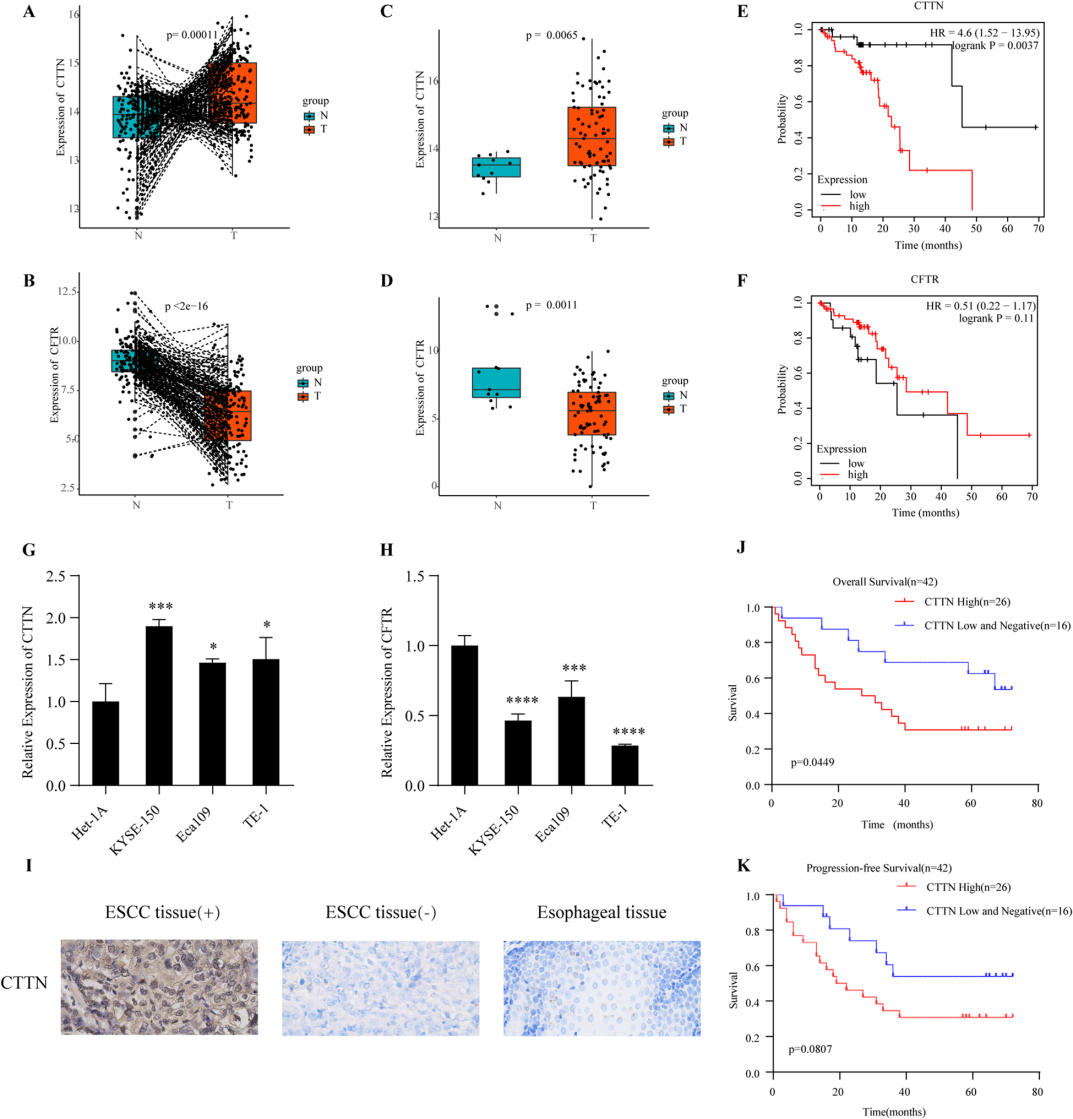
Multiple verifications of CTTN and CFTR expression in ESCC. (**A**, **B**) The expression patterns of CTTN and CFTR in GSE53625 (ESCC tissue vs paired normal tissues (n = 179)). (**C**, **D**) The expression patterns of CTTN and CFTR in TCGA-ESCC cohort (ESCC tissue (n = 81) vs normal tissues (n = 11)). (**E**, **F**) Kaplan–Meier survival analysis of OS in ESCC patients from Kaplan–Meier Plotter database based on CTTN expression or CFTR expression (n = 81). (**G**, **H**) Relative expression of CTTN and CFTR in ESCC cell lines (KYSE-150, Eca109 and TE-1) and normal esophageal epithelial cells (Het-1A) by qRT-PCR. (*P < 0.05; **p < 0.01; ***p < 0.001). (**I**) Immunohistochemistry staining was performed to detect the expression of CTTN in ESCC tissues and normal esophageal tissues (original magnification,·× 400). (**J**, **K**) Overall survival and progression-free survival of ESCC patients were estimated by the Kaplan–Meier survival analysis according to CTTN expression levels.

Among the 8 ARGs in the prognostic signature, 2 genes (CTTN and CFTR) were identified as differentially expressed genes between tumors and normal tissues in the GSE53625 dataset (Fig. 9A, B). To reduce bias, we used a multi-database approach to determine the expression of 8 ARGs at the tissue and cell levels (Supplementary Table 10). The expression differences of CTTN and CFTR in TCGA and Oncomine databases were completely consistent with the above analysis, which showed that CTTN was up-regulated in ESCC, while CFTR was down-regulated in ESCC (Fig. 9C, D, Supplementary Fig. 3). In the Cancer Cell Line Encyclopedia database, CTTN was highly expressed in ESCC cell line, while CFTR was lowly expressed in ESCC cell line, which was consistent with the above differential expression pattern (Supplementary Fig. 4). In addition, the results of Kaplan–Meier Plotter database indicated that high expression level of CTTN was significantly associated with poor OS in ESCC patients (Fig. 9E), while no statistical difference was shown between CTFR expression and OS in ESCC patients (Fig. 9F). The remaining six genes in the prognostic signature had similar expression patterns in the four databases mentioned above (Supplementary Fig. 5).

### Preliminary experimental validation

In order to verify the accuracy of bioinformatics analysis, we used ESCC cell line and ESCC tissue to detect the expression level of ARGs in prognostic signature. The qRT-PCR results were consistent with the above bioinformatics analysis. Compared with human normal esophageal epithelial cells (Het-1A), CTTN was increased and CFTR was down-regulated in ESCC cell lines (Fig. 9G, H). At the cellular level, the expression patterns of the other six genes were similar to those of previous bioinformatics analysis (Supplementary Fig. 6). To further study the CTTN protein expression, we performed immunohistochemistry staining of 42 ESCC tumor samples and 10 normal esophageal samples. The results indicate the expression of CTTN in tumor tissues was significantly higher than that of normal esophageal tissues (Fig. 9I, Supplementary Table 11). Moreover, Kaplan–Meier survival analysis showed that OS and progression-free survival were shorter in the group with high CTTN than in the group with low- negative CTTN (Fig. 9J, K).

## Discussion

In China, ESCC is the most common pathological subtype, and patients with ESCC have a poor prognosis due to unknown pathogenesis. Therefore, it is urgent to explore new and accurate molecular biomarkers for prognosis. Autophagy plays a dual role in tumors by inhibiting tumor growth or promoting tumor invasion, and has been reported to be involved in the diagnosis and treatment of esophageal squamous cell carcinoma^8,9,31^. Therefore, it is of great significance to search for biomarkers related to autophagy for individualized treatment and prognosis of ESCC patients.

In this study, we identified 87 differentially expressed ARGs by integrating autophagy-related genes from HADB and Molecular Signatures Database based on ESCC expression profile from GEO data. GO and KEGG analysis of differentially expressed ARGs showed that they were mainly involved in the regulation of autophagy, TNF signaling pathway and Apoptosis signaling pathway. We then used univariate Cox regression analysis to identify prognosis-related ARGs. Furthermore, a novel prognostic signature containing eight autophagy-related genes was established, which showed good prognostic performance and was a significant independent prognostic factor for predicting the long-term outcomes in patients with ESCC. Correlation analysis showed that the risk score of autophagy-related signature was associated with survival outcomes, tumor stage, and N stage. The GSEA results revealed the prognostic signature may be involved in the cancer- and autophagy-related signaling pathways and immunoregulatory signaling pathways. Notably, we also verified ARGs expression using ESCC cell lines, ESCC tissues and multiple databases. In summary, our research demonstrated that autophagy dysfunction was important during the process of ESCC. Most importantly, for the first time, we developed a robust ESCC prognostic risk signature based on ARGs, which may provide a more accurate assessment of the prognosis of ESCC patients and a more personalized treatment strategy.

It has been reported that most of the 8 ARGs (VIM, UFM1, TSC2, SRC, MEFV, CTTN, CFTR and CDKN1A) are related to cancer. VIM is an important marker of epithelial-mesenchymal transition (EMT) that plays a key role in the progression and metastasis of a variety of tumors, including ESCC^32^. UFM1 is a potential marker protein that induces DNA double-strand breaks response, and is associated with breast cancer progression^33,34^. TSC2 is a negative regulator upstream of mTOR, and the TSC2-mTOR signaling pathway plays a crucial role in the regulation of tumor autophagy^35^. SRC was a prognostic factor for overall survival in patients with colorectal cancer and glioblastoma multiforme^36,37^. CTTN was closely correlated with the prognosis of head and neck cancer and glioma^38,39^. It has been reported that CFTR can be used as a prognostic biomarker of nasopharyngeal carcinoma, colorectal cancer and head and neck cancer^40–42^. CDKN1A has been reported to play a protective role in the prognosis of cervical cancer^43^. At present, the mechanism of these eight genes in ESCC has not been fully elucidated.

CIBERSORT analysis showed that the risk score was correlated with TICs, including B cells naive, Plasma cells, T cells CD4 memory resting and dendritic cells resting. Meanwhile, the mRNA expression levels of PD-1and PD-L1were negatively correlated with the risk score. These data suggest further studies will be interesting to explore specific mechanisms of risk signature and tumor-infiltrating immune cells and ARGs-related risk score may provide potential guidance for personalized treatments of ESCC.

Ferroptosis is a newly introduced form of programmed cell death discovered in recent years. Accumulating studies have revealed Ferroptosis could inhibit tumor growth or promote tumor proliferation in tumor development, and there is crosstalk with autophagy at the molecular level^22,23^ Ferroptosis-related genes, such as GPX4 and HMOX1, were related to prognosis to the prognosis of esophageal squamous cell carcinoma^44^. In the present study, we found that more than 1/3 of the ferroptosis-related genes (33.97%, (71/209)) were associated with the risk score, which further provided more information for targeting ferroptosis therapy.

Nonetheless, there are still some limitations in this study. First, although we have used the bootstrap method to verify the robustness of the prognostic signature established in 179 ESCC patients, external datasets are still needed to further validate the prognostic signature. Second, although we performed qPCR and immunohistochemistry analysis, the role and mechanism of genes that constitute the prognostic signature of esophageal squamous cell carcinoma need to be explored deeply.

In brief, we constructed an autophagy-related prognostic signature for ESCC for the first time and successfully verified its reliability. Our study might provide a novel approach for effective prediction of clinical outcomes and individualized therapeutic strategies selection.

## Supplementary Information

Supplementary Information.

## Data Availability

Publicly available datasets were analyzed in this study. This data can be found at: https://www.ncbi.nlm.nih.gov/geo/query/acc.cgi.

## References

[1] Bray F (2018). Global cancer statistics 2018: GLOBOCAN estimates of incidence and mortality worldwide for 36 cancers in 185 countries. CA Cancer J. Clin..

[2] Malhotra GK (2017). Global trends in esophageal cancer. J. Surg. Oncol..

[3] Chen W (2016). Cancer statistics in China, 2015. CA Cancer J. Clin..

[4] Huang F-L, Yu S-J (2018). Esophageal cancer: Risk factors, genetic association, and treatment. Asian J. Surg..

[5] Fitzmaurice C (2015). The global burden of cancer 2013. JAMA Oncol..

[6] Parzych KR, Klionsky DJ (2014). An overview of autophagy: Morphology, mechanism, and regulation. Antioxid. Redox Signal..

[7] Amaravadi R, Kimmelman AC, White E (2016). Recent insights into the function of autophagy in cancer. Genes Dev..

[8] Langer, R., Streutker, C. J. & Swanson, P. E. in *13th Oeso World Conference: The Esophagiome Ii* Vol. 1381 *Annals of the New York Academy of Sciences* (eds R. Giuli & H. Gregersen) 113–121 (2016).

[9] Khan T (2020). Autophagy modulators for the treatment of oral and esophageal squamous cell carcinomas. Med. Res. Rev..

[10] Li J (2014). LncRNA profile study reveals a three-lncRNA signature associated with the survival of patients with oesophageal squamous cell carcinoma. Gut.

[11] Ritchie ME (2015). limma powers differential expression analyses for RNA-sequencing and microarray studies. Nucleic Acids Res..

[12] Carbon S (2019). The Gene Ontology Resource: 20 years and still GOing strong. Nucleic Acids Res..

[13] Yu G, Wang L-G, Han Y, He Q-Y (2012). clusterProfiler: An R package for comparing biological themes among gene clusters. Omics J. Integr. Biol..

[14] Kanehisa M, Goto S (2000). KEGG: Kyoto Encyclopedia of Genes and Genomes. Nucleic Acids Res..

[15] Kanehisa M (2019). Toward understanding the origin and evolution of cellular organisms. Protein Sci..

[16] Kanehisa M, Furumichi M, Sato Y, Ishiguro-Watanabe M, Tanabe M (2021). KEGG: Integrating viruses and cellular organisms. Nucleic Acids Res..

[17] KEGG (Kyoto Encyclopedia of Genes and Genomes). www.kegg.jp/kegg/kegg1.html.

[18] Wang J (2019). Prognostic nomogram based on immune scores for breast cancer patients. Cancer Med..

[19] Li J-P (2020). A seven immune-related lncRNAs model to increase the predicted value of lung adenocarcinoma. Front. Oncol..

[20] Thorsson V (2018). The immune landscape of cancer. Immunity.

[21] Newman AM (2019). Determining cell type abundance and expression from bulk tissues with digital cytometry. Nat. Biotechnol..

[22] Jiang M (2020). Targeting ferroptosis for cancer therapy: Exploring novel strategies from its mechanisms and role in cancers. Transl. Lung Cancer Res..

[23] Zhou Y (2019). The crosstalk between autophagy and ferroptosis: What can we learn to target drug resistance in cancer?. Cancer Biol. Med..

[24] Rhodes DR (2007). Oncomine 3.0: Genes, pathways, and networks in a collection of 18,000 cancer gene expression profiles. Neoplasia.

[25] Ghandi M (2019). Next-generation characterization of the cancer cell line encyclopedia. Nature.

[26] Shi X (2020). Genome-wide analysis of lncRNAs, miRNAs, and mRNAs forming a prognostic scoring system in esophageal squamous cell carcinoma. PeerJ.

[27] Nagy A, Munkacsy G, Gyorffy B (2021). Pancancer survival analysis of cancer hallmark genes. Sci. Rep..

[28] Sun Y (2018). LINC00657 played oncogenic roles in esophageal squamous cell carcinoma by targeting miR-615-3p and JunB. Biomed. Pharmacother..

[29] Pan SP (2018). Lobaplatin promotes radiosensitivity, induces apoptosis, attenuates cancer stemness and inhibits proliferation through PI3K/AKT pathway in esophageal squamous cell carcinoma. Biomed. Pharmacother..

[30] R Core Team. R: A language and environment for statistical computing. R Foundation for Statistical Computing, Vienna, Austria. http://www.R-project.org/ (2020).

[31] Kimmelman AC, White E (2017). Autophagy and tumor metabolism. Cell Metab..

[32] Hu HF (2020). Identification of miR-515-3p and its targets, vimentin and MMP3, as a key regulatory mechanism in esophageal cancer metastasis: Functional and clinical significance. Signal Transduct Target Ther.

[33] Yoo HM (2014). Modification of ASC1 by UFM1 is crucial for ER alpha transactivation and breast cancer development. Mol. Cell.

[34] Fang Z, Pan Z (2019). Essential role of ubiquitin-fold modifier 1 conjugation in DNA damage response. DNA Cell Biol..

[35] Ranek MJ (2019). PKG1-modified TSC2 regulates mTORC1 activity to counter adverse cardiac stress. Nature.

[36] Cirotti C, Contadini C, Barila D (2020). SRC kinase in Glioblastoma: News from an Old Acquaintance. Cancers (Basel).

[37] Jin W (2020). Regulation of Src family kinases during colorectal cancer development and its clinical implications. Cancers (Basel).

[38] Ramos-Garcia P (2019). Prognostic and clinicopathological significance of CTTN/cortactin alterations in head and neck squamous cell carcinoma: Systematic review and meta-analysis. Head Neck J. Sci. Spec. Head Neck.

[39] Zhang S, Qi Q (2015). MTSS1 suppresses cell migration and invasion by targeting CTTN in glioblastoma. J. Neurooncol..

[40] Tu Z (2016). CFTR is a potential marker for nasopharyngeal carcinoma prognosis and metastasis. Oncotarget.

[41] Shin Y (2020). Epigenetic modification of CFTR in head and neck cancer. J. Clin. Med..

[42] Than BLN (2017). CFTR is a tumor suppressor gene in murine and human intestinal cancer. Oncogene.

[43] Zhang X, Li F, Zhu L (2018). Clinical significance and functions of microRNA-93/CDKN1A axis in human cervical cancer. Life Sci..

[44] Shishido Y (2020). Antitumor effect of 5-aminolevulinic acid through ferroptosis in esophageal squamous cell carcinoma. Ann. Surg. Oncol..

