# Supplementary material for: Identification and validation of an autophagy-related gene signature for predicting prognosis in patients with esophageal squamous cell carcinoma
Source: Sci Rep. 2022 Feb 4;12:1960. doi: 10.1038/s41598-022-05922-4 (PMC8817038; doi:10.1038/s41598-022-05922-4)
Supplement: Supplementary file 1 — Supplementary Information. [file 41598_2022_5922_MOESM1_ESM.zip › Supplementary material/Supplementary_figure .docx]

Supplementary Figures
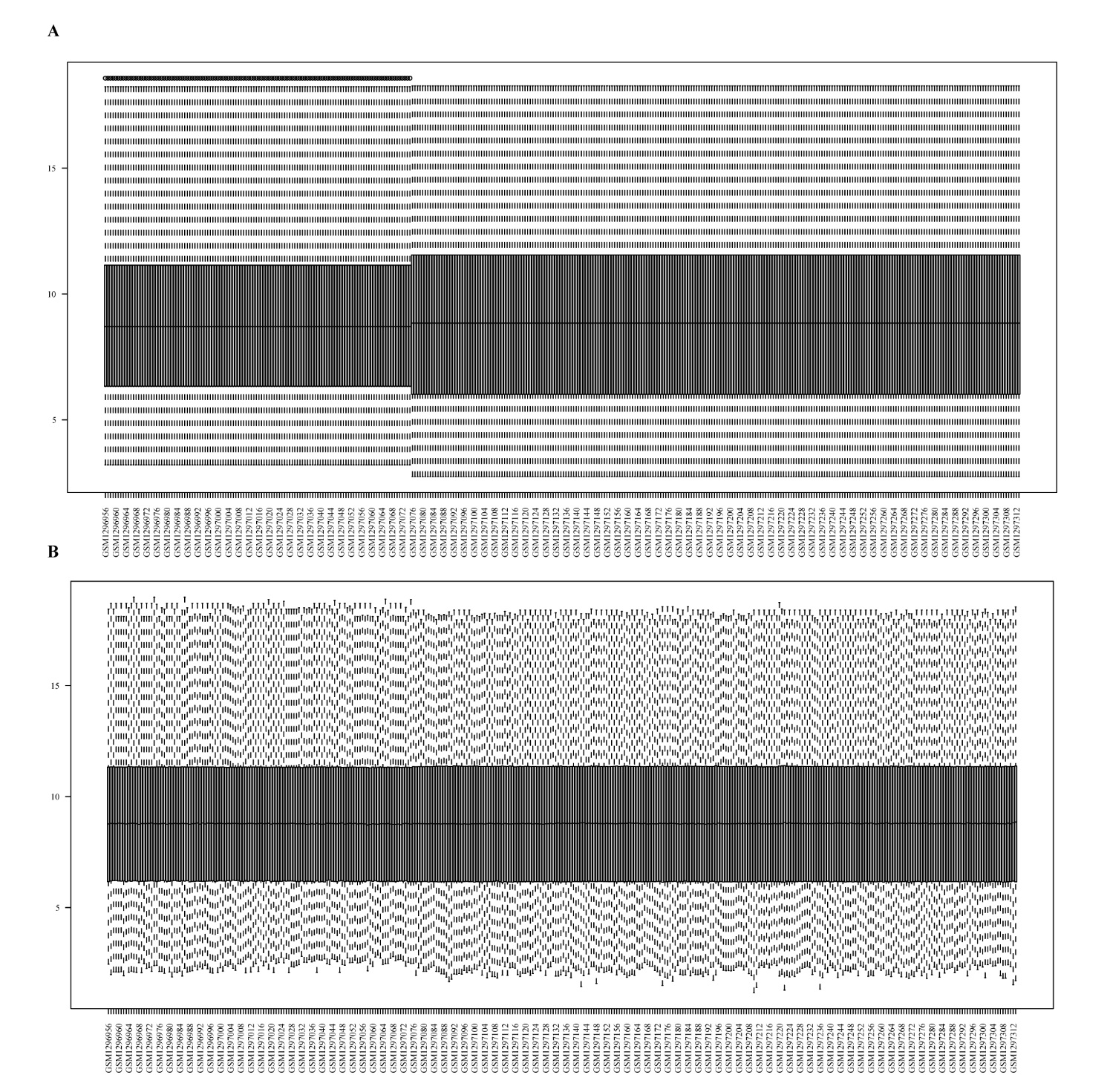


**Supplementary Figure 1.** The RNA expression profiles of GSE53625 dataset. (A) Batch effect existence (B) Batch effect correction

**
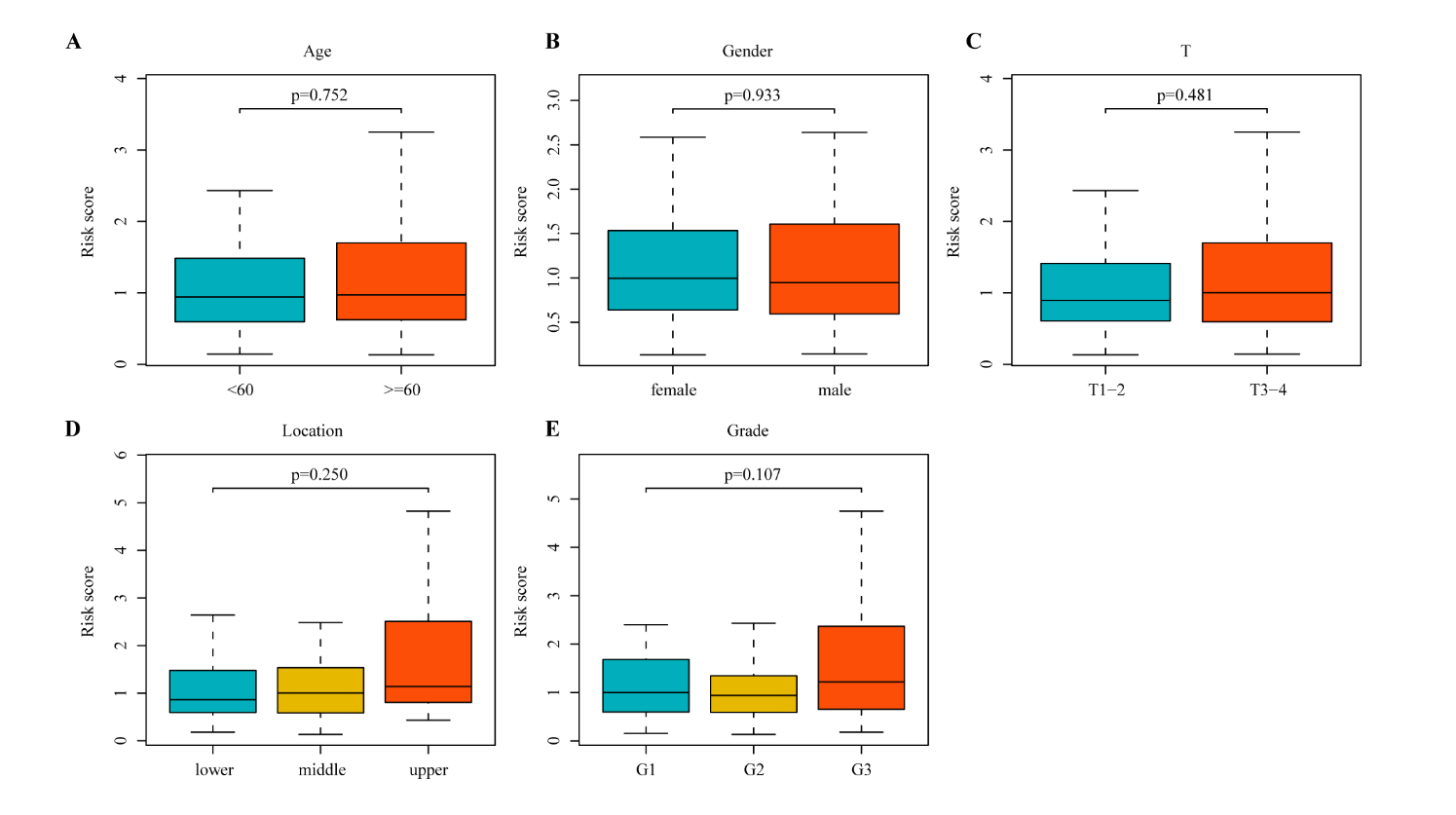
**

**Supplementary Figure 2.** Clinical significance of the prognostic signature of ESCC. (A) age; (B) gender; (C) T stage; (D) location; (E) grade


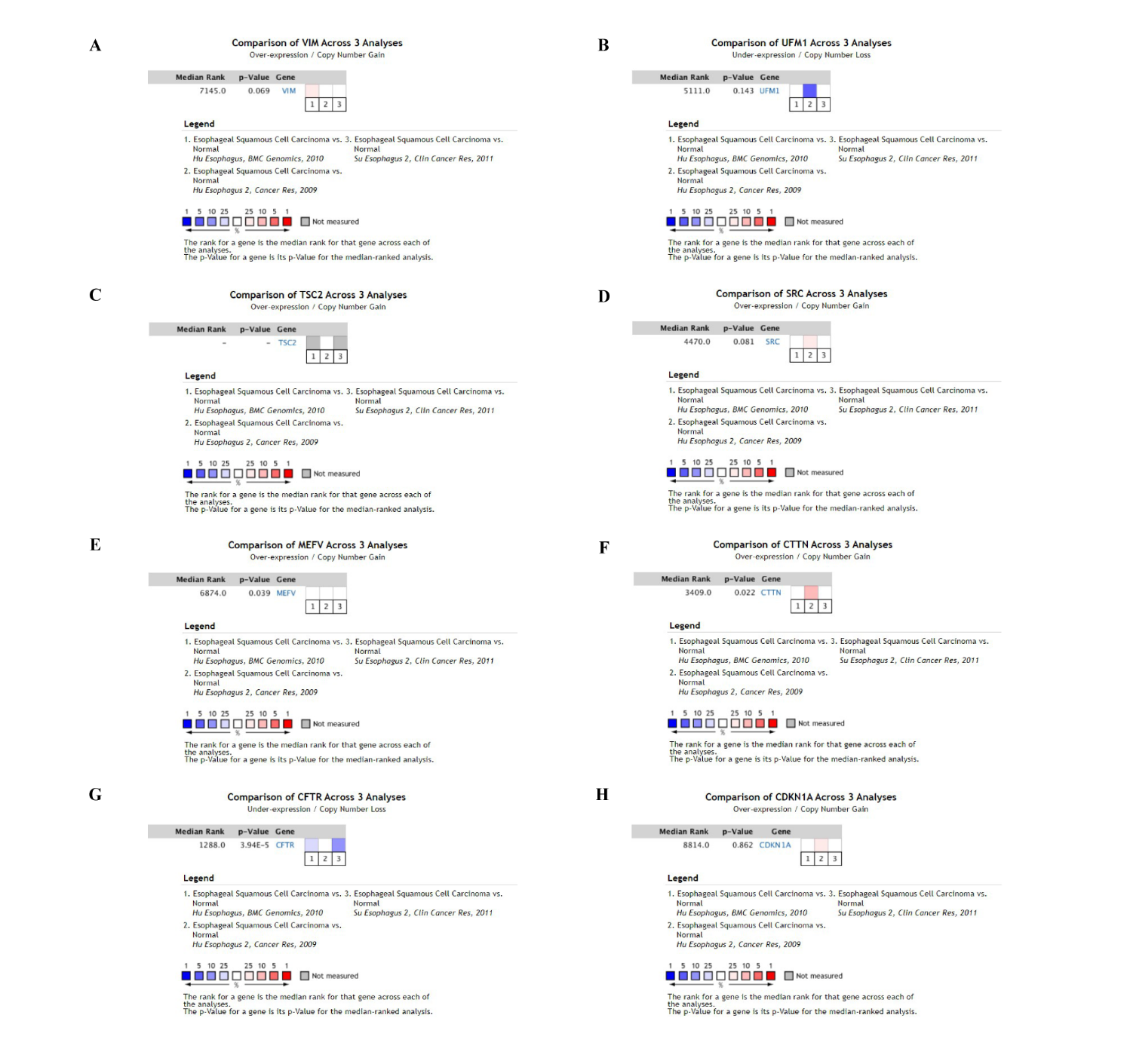


**Supplementary Figure 3.** The expression of VIM (A), UFM1 (B), TSC2 (C), SRC (D), MEFV (E), CTTN (F), CFTR (G) and CDKN1A (H) in multiple ESCC tumor studies was validated at the transcriptomic level using the Oncomine database.


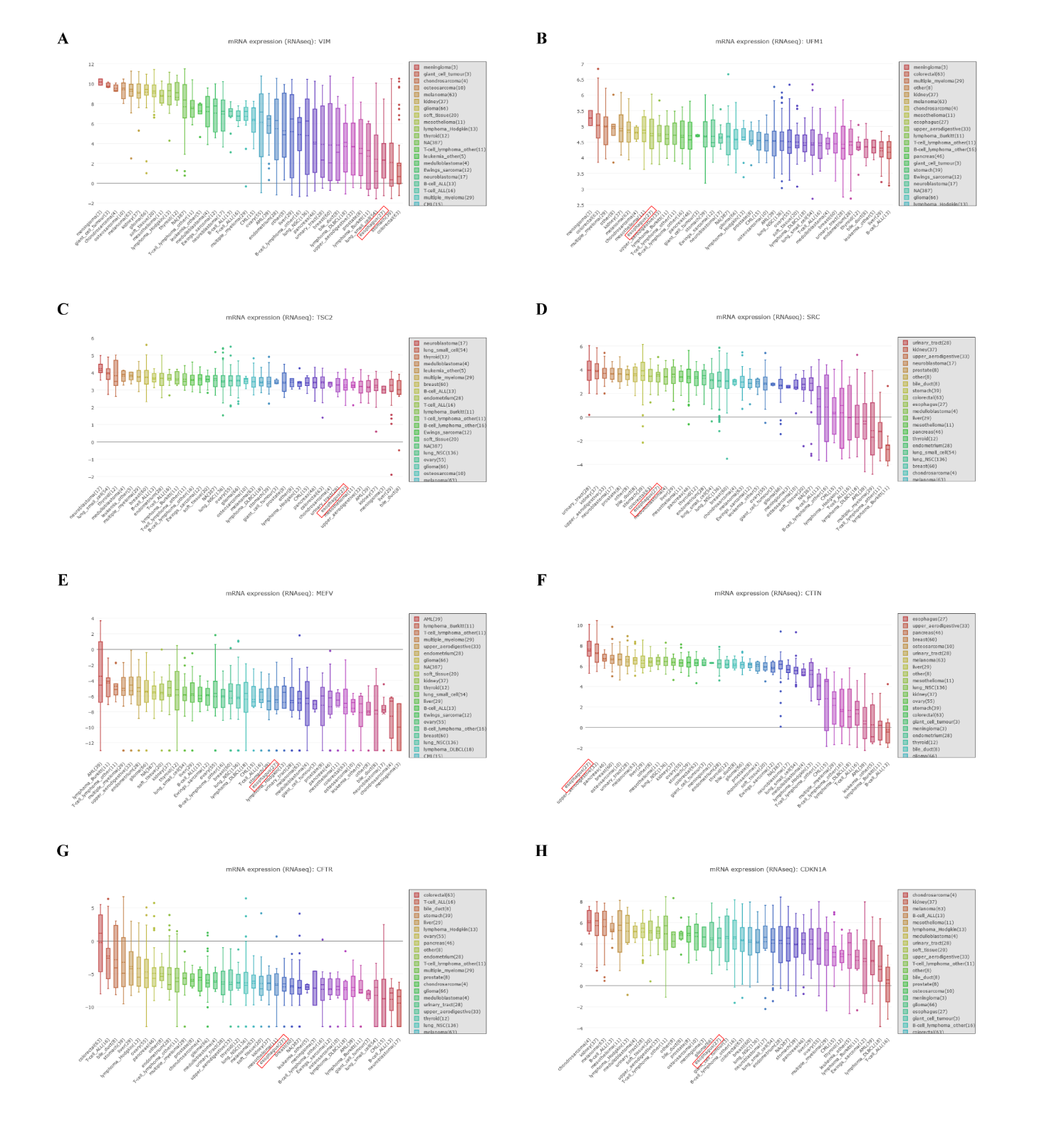


**Supplementary Figure 4.** The expression levels of VIM (A), UFM1 (B), TSC2 (C), SRC (D), MEFV (E), CTTN (F), CFTR (G) and CDKN1A (H) in various tumor cell lines in the Cancer Cell Line Encyclopedia (CCLE).


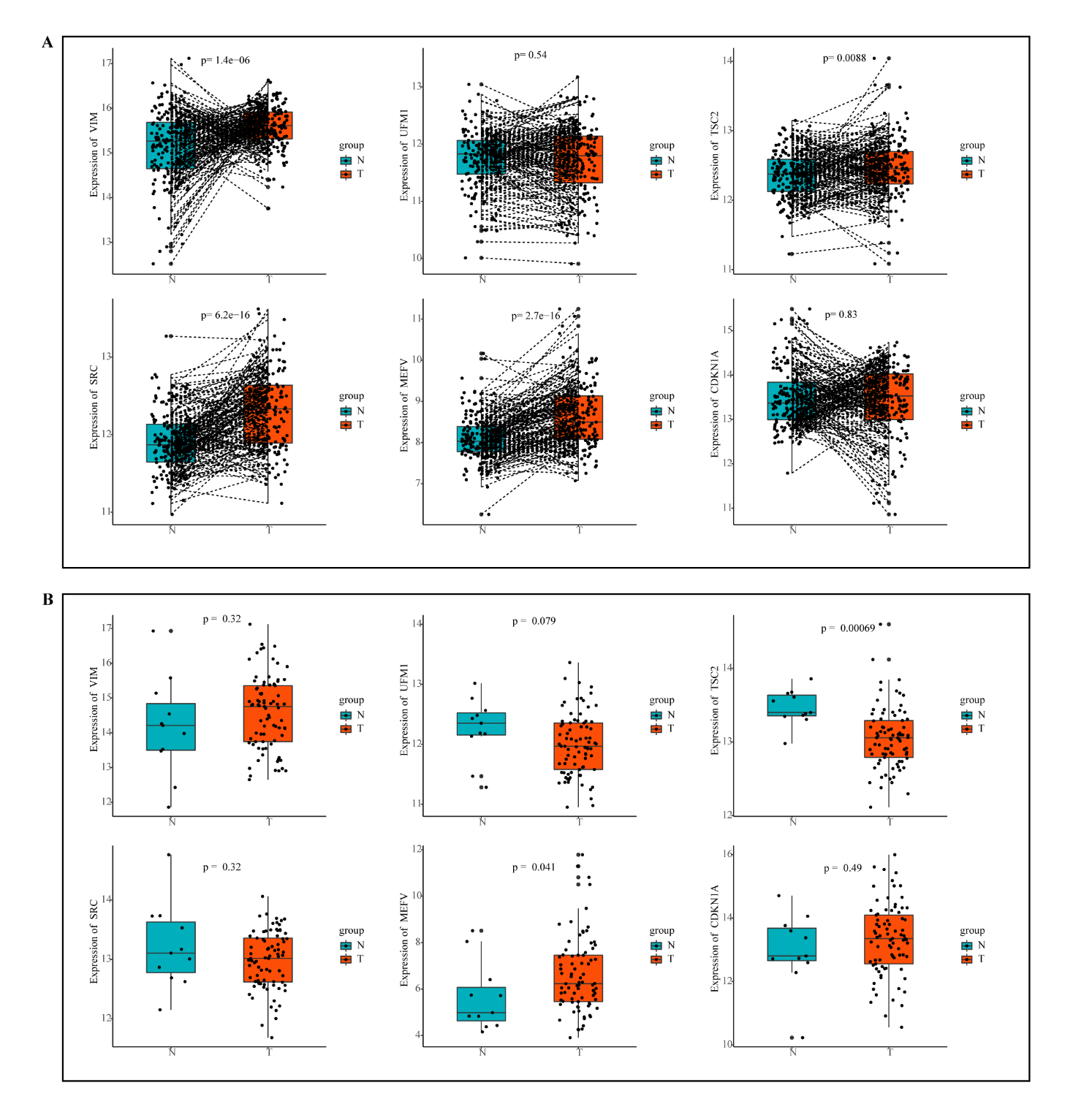


**Supplementary Figure 5.** The expression patterns of remaining the six genes (VIM, UFM1, TSC2, SRC, MEFV and CDKN1A) in GSE53625 and TCGA database. (A) The expression patterns of the six genes in GSE53625. (B) The expression patterns of the six genes in TCGA-ESCC cohort.


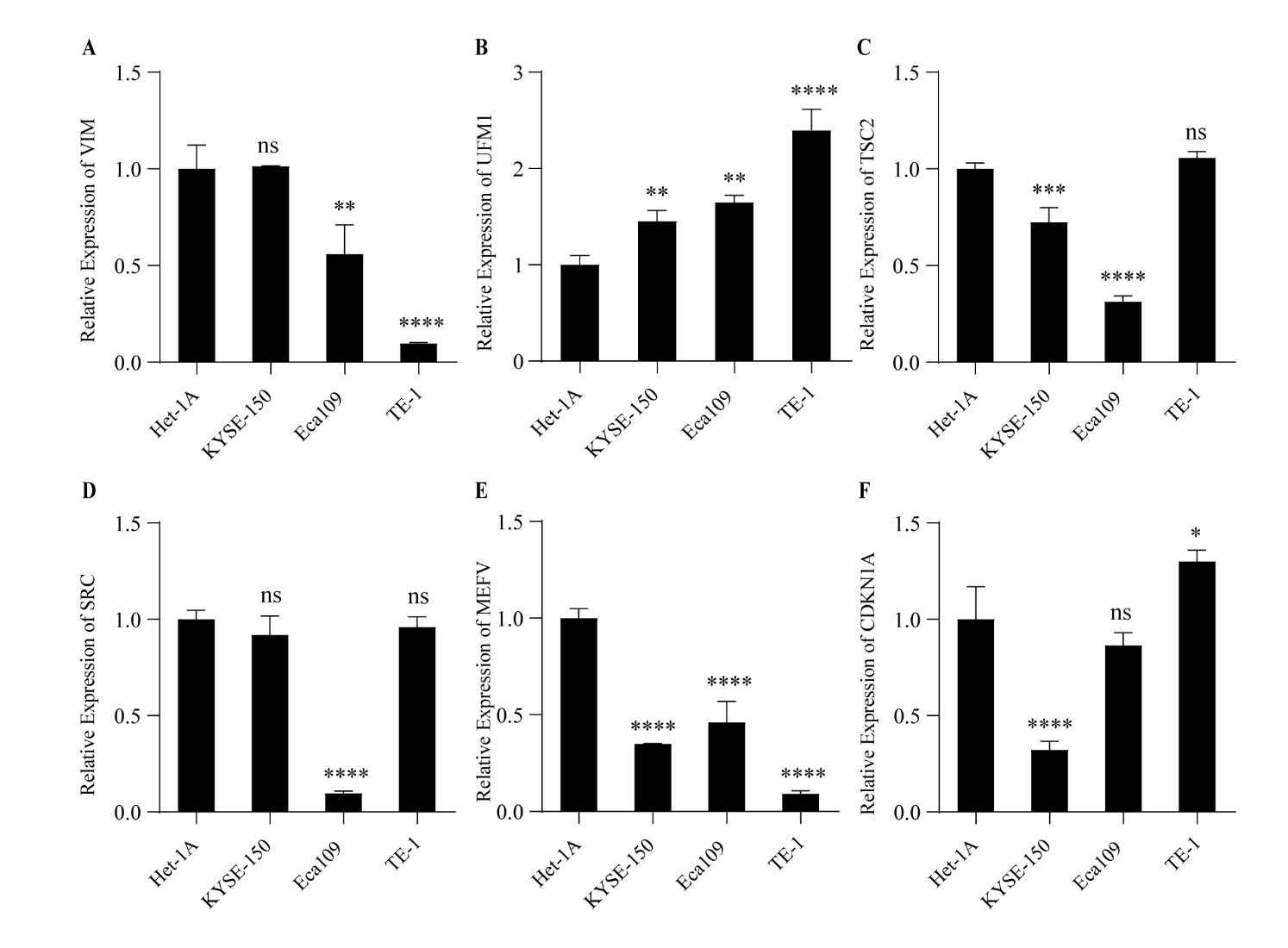


**Supplementary Figure 6.** Relative expression of VIM (A), UFM1 (B), TSC2 (C), SRC (D), MEFV (E), and CDKN1A (F) in ESCC cell lines (KYSE-150, Eca109 and TE-1) and normal esophageal epithelial cells (Het-1A) by qRT -PCR. (* P < 0.05, ** p < 0.01, *** p < 0.001, **** p < 0.0001, ns not significant).
